# Drunkorexia: an empirical investigation of disordered eating in direct response to saving calories for alcohol use amongst Australian female university students

**DOI:** 10.1186/2050-2974-1-S1-P6

**Published:** 2013-11-14

**Authors:** Alissa Knight, Susan Simpson

**Affiliations:** 1 The University of South Australia, Australia

## Introduction

The term "Drunkorexia" has been used by the popular media to denote the use of extreme weight-control behaviours to compensate for planned binge drinking. This hazardous phenomenon has been declared a genuine public health concern due to its rapid growth as a popular trend among Australian female university students. To date, no empirical studies have explored the prevalence of Drunkorexia in an Australian sample.

## Research aim

The present study aimed to address this gap by conducting systematic investigation of the phenomenon of Drunkorexia in a population of non-clinical Australian undergraduate female university students.

## Method

One hundred and thirty nine healthy female Australian undergraduate university students aged between 18-29 years; (M=21.4, SD=2.80) completed the self-report Compensatory Eating and Behaviors in Response to Alcohol Consumption Scale (CEBRACS) to screen for Drunkorexia symptomatology.

## Results

In the sample tested, over 79.1% (n = 110) of the participants reported engaging in characterised Drunkorexia behaviour. In addition, as predicted, series of bivariate Pearson correlations, and hierarchical multiple regression analyses indicated that binge drinking, group social norm of thinness, and group social norm of drinking were positively related to total Drunkorexia behaviour.

## Discussion

The findings of this study provide preliminary empirical evidence that Australian female university students are engaging in Drunkorexia behaviours, and have far reaching theoretical and clinical implications for the area, and for the health and well-being of Australian female university students.

